# Synchronous Symmetry Breaking in Neurons with Different Neurite Counts

**DOI:** 10.1371/journal.pone.0054905

**Published:** 2013-02-11

**Authors:** Zachary D. Wissner-Gross, Mark A. Scott, Joseph D. Steinmeyer, Mehmet Fatih Yanik

**Affiliations:** 1 Department of Physics, Harvard University, Cambridge, Massachusetts, United States of America; 2 Division of Health, Science, and Technology, Massachusetts Institute of Technology, Cambridge, Massachusetts, United States of America; 3 Department of Electrical Engineering and Computer Science, Massachusetts Institute of Technology, Cambridge, Massachusetts, United States of America; 4 Department of Biological Engineering, Massachusetts Institute of Technology, Cambridge, Massachusetts, United States of America; University of Michigan, United States of America

## Abstract

As neurons develop, several immature processes (i.e., neurites) grow out of the cell body. Over time, each neuron breaks symmetry when only one of its neurites grows much longer than the rest, becoming an axon. This symmetry breaking is an important step in neurodevelopment, and aberrant symmetry breaking is associated with several neuropsychiatric diseases, including schizophrenia and autism. However, the effects of neurite count in neuronal symmetry breaking have never been studied. Existing models for neuronal polarization disagree: some predict that neurons with more neurites polarize up to several days later than neurons with fewer neurites, while others predict that neurons with different neurite counts polarize synchronously. We experimentally find that neurons with different neurite counts polarize synchronously. We also show that despite the significant differences among the previously proposed models, they all agree with our experimental findings when the expression levels of the proteins responsible for symmetry breaking increase with neurite count. Consistent with these results, we observe that the expression levels of two of these proteins, HRas and shootin1, significantly correlate with neurite count. This coordinated symmetry breaking we observed among neurons with different neurite counts may be important for synchronized polarization of neurons in developing organisms.

## Introduction

As neurons develop, immature processes known as neurites grow out of the cell body and mature into either axons or dendrites. Each of these neurites is initially capable of becoming an axon [1], [2], but after 24–48 h in culture, typically only one of the neurites becomes the axon, while the others become dendrites [3]–[5]. The polarization of neurons is essential for the proper wiring of the nervous system, and abnormal polarization is associated with several neuropsychiatric diseases, including schizophrenia [6] and autism [7], [8]. The precise mechanism of this neuronal symmetry breaking remains an open question, but several pathways in the process have recently been elucidated [9]–[15].

However, the role of neurite count in neuronal symmetry breaking has never been examined, despite the fact that neurite count can vary significantly, and is one of the most salient properties of developing neurons. Leading biophysical models suggest that neurites compete for specific proteins (such as HRas and shootin1) in a winner-take-all fashion during axon specification [10], [16]–[18].

These biophysical models include the Samuels model [17], the Fivaz model [10], and the Toriyama model [18]. The Samuels model was the first published model to describe axon specification using the transport and diffusion of a rate-limiting chemical for neurite growth. The Fivaz and Toriyama models are more recent, and similarly involve competition among neurites for a pool of proteins involved in neuronal polarization (HRas in the Fivaz model, shootin1 in the Toriyama model).

Since the number of interactions among competing neurites and the overall complexity of the neuron should increase with neurite count, one might expect a neuron with more neurites to polarize more slowly. Indeed, such a finding has been previously reported in these models. For example, in the Toriyama model, neurons with 10 neurites are predicted to polarize several days later than neurons with only 3 neurites [18]. However, previous experiments have shown that neurons with varying final neurite counts all polarize within the same 48 h time window [3]–[5]. Thus, there remains a fundamental disagreement between the theoretical models and the experimentally observed biology.

In this work, we first showed that neurons with different neurite counts polarize synchronously. We then both experimentally and computationally investigated the mechanism underlying this phenomenon, and offer simple modifications to the existing models so that they correctly predict rates of symmetry breaking that are independent of neurite count.

## Results

### Neurons with Different Neurite Counts Polarize Synchronously

**Figure 1:**
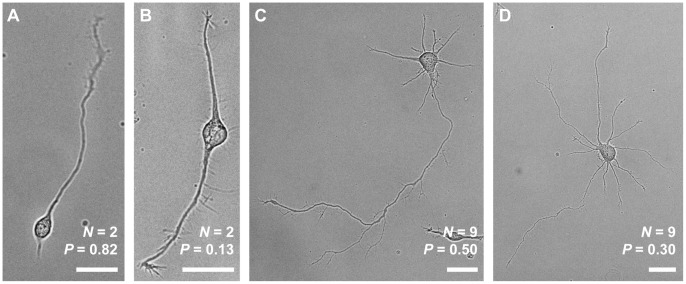
Bright-field micrographs of four different neurons. Neurite count (*N*) and polarity (*P*) using Eq. (1) are also indicated for each neuron. **A** and **B** show examples of neurons with two neurites that are relatively more (**A**) or less (**B**) polarized. **C** and **D** similarly show neurons with many neurites that are relatively more (**C**) or less (**D**) polarized. The image in **A** was taken 28.5 h after plating, and **B**–**D** were taken at 52.5 h after plating. All scale bars are 25 µm.

We measured neurite lengths and polarities in two hundred E18 rat hippocampal neurons, cultured on glass coated with poly-D-lysine and laminin, at nine different time points over the course of two days. We introduce the following metric to quantify neuronal polarity of neurons with multiple neurites (see Methods):
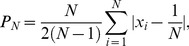
(1)where *N* is the neurite count, and *x­_i_* is the relative length of neurite *i*. Fig. 1 shows four different neurons, as well as their neurite counts and their polarities calculated using Eq. (1). For a discussion and justification of this metric, see the Materials and Methods section. We also verified that the neurons were functionally polarizing over this time scale by performing an immunocytochemical stain for axonal and dendritic markers (Fig. S1).

**Figure 2:**
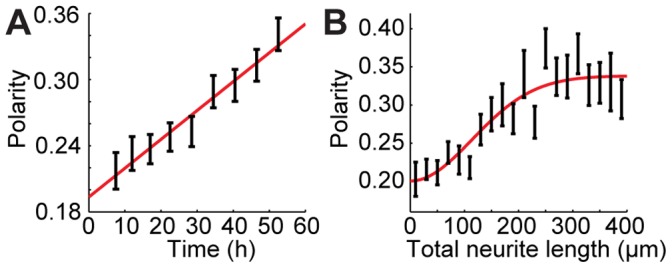
Dynamics of neuron polarization as defined by Eq. (1). **A**, Polarity versus time. The solid line is a linear fit to the data. **B**, Polarity versus total neurite length. Total neurite length was binned at intervals of 20 µm. The solid line is a Gaussian fit to the data, with an inflection point at 114 µm suggesting a phase transition between unpolarized and polarized states. The nonlinearity of this data agrees with previous work on the polarization of neurons with exactly two neurites [19].

Using Eq. (1), we observed an increasing polarity as a function of time (*p* < 10^−10^ by Pearson correlation, Fig. 2A), as well as the expected phase transition in polarity as total neurite length exceeded approximately 100 µm (Fig. 2B), consistent with our previously reported results in neurons with exactly two neurites [19]. The specific number of neurons at each time point can be found in Table S1.

Just as the average polarity increased over time, we found that the number of neurons whose polarity exceeded different thresholds also increased over time. For example, the fraction of neurons whose polarity exceeded 0.3 increased from 0.21 at 7.5 h, to 0.40 at 40.5 h, to 0.57 at 52.5 h.

**Figure 3:**
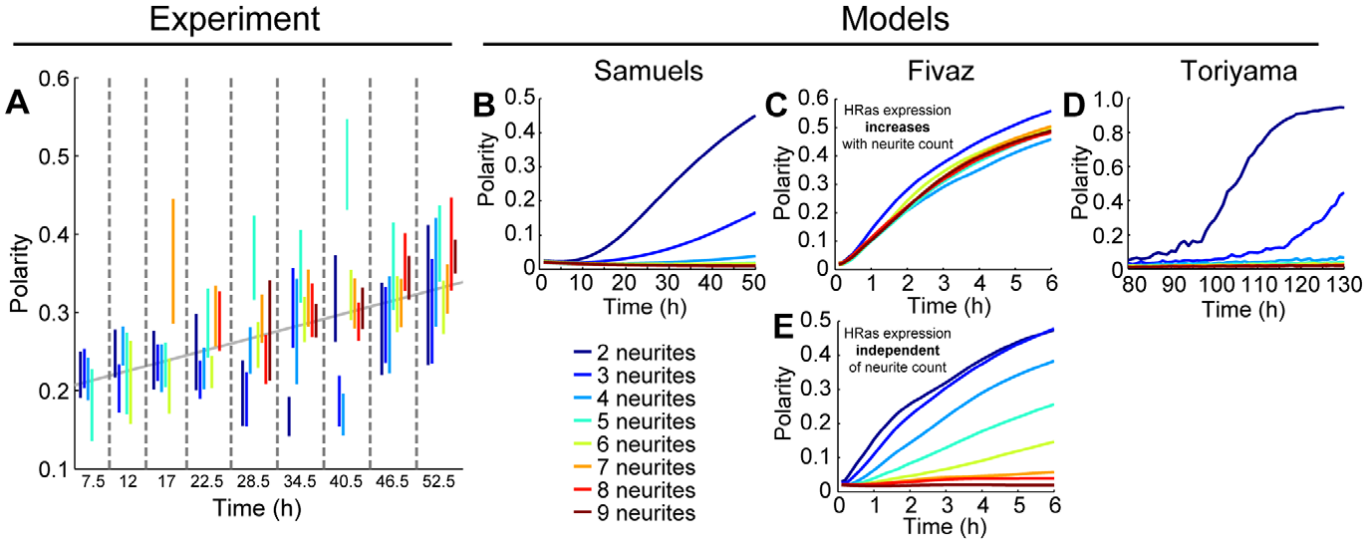
Experimentally and theoretically predicted symmetry breaking in neurons with different neurite counts. Each color series indicates a different neurite count, as listed in the legend. **A**, Experimental measurements of neuronal polarity as a function of time and neurite count. Dashed lines separate data from different discrete time points. Bars indicate mean plus/minus SE, and neurite counts at each time point are only shown if at least 3 neurons had that neurite count at that time point. **B**–**D**, Computationally predicted polarity vs. time curves for neurons with different neurite counts using the Samuels (**B**), Fivaz (**C**), and Toriyama (**D**) models. **E**, Computationally predicted polarity vs. time curves for neurons with different neurite counts using a modified version of the Fivaz model in which HRas expression is independent of neurite count. The original Fivaz model, in which HRas expression increases with neurite count, is shown in **C**.

We next experimentally determined whether neurons with different neurite counts polarize synchronously or asynchronously. At each time point, with the lone exception at 40.5 h after plating, we observed no statistical difference among the polarities of neurons with different neurite counts (*p* > 0.05 by ANOVA, Fig. 3A). We also found that there was no significant correlation (*p* > 0.01 by Pearson correlation) between neurite count and polarity at any of the time points, including the 40.5 h time point.

We compared these experimental results with those predicted by the Samuels, Fivaz, and Toriyama models for symmetry breaking in developing neurons. In particular, we found that the Samuels and Toriyama models significantly disagreed with our measurements. While neurons with each neurite count will ultimately polarize in these two models, neurons with many neurites are predicted to begin polarizing up to several days later than neurons with fewer neurites, as indicated by the large gaps among the polarity vs. time curves in Figs. 3B and 3D. The Fivaz model, on the other hand, predicted that neurite count had little effect on polarization, as illustrated by the close proximity of the polarity vs. time curves in Fig. 3C. Thus, the Fivaz model was unique among the three models in its agreement with our experimental data.

Another unique aspect of the Fivaz model is that it assumes that total HRas expression levels within the neuron increase with neurite count. The Toriyama model, however, assumes shootin1 expression levels are independent of neurite count. Similarly, the Samuels model assumes that the quantity of the rate-limiting chemical for neurite growth has no dependence on neurite count.

We suspected whether the correlation between HRas expression and neurite count in the Fivaz model may be necessary for the synchronous polarization of neurons with different neurite counts in that model. By normalizing HRas expression levels so that they were effectively the same for all neurons, we found that neurons with different neurite counts polarized asynchronously (Fig. 3E), as in the Samuels and Toriyama models.

### Neurite Count is Dynamic Throughout Polarization

**Figure 4:**
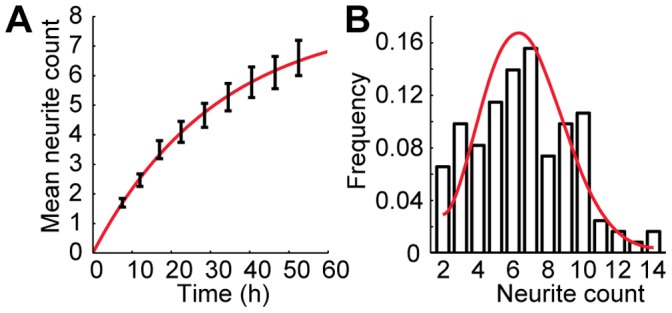
Neurite sprouting in developing neurons. **A**, Mean neurite count as a function of time. The solid line is an exponential fit to the data, constrained to include the origin. Error bars are SE. **B**, Neurite count distribution at the final measured time point 52.5 h after plating. The solid line is a simulated distribution using the exponential fit from **A** to calculate the sprouting rate and assumes random neurite sprouting independent of neurite count. Error bars are SE.

The Samuels, Fivaz, and Toriyama models all assume that neurite count does not change throughout the polarization process. However, we observed that new neurites sprouted while the neurons were breaking symmetry. We specifically found that neurite sprouting occurred in an exponentially decaying fashion, with a characteristic time of approximately 32 h and an asymptotic mean neurite count of approximately 8.0 (Fig. 4A).

We further analyzed the neurite count distribution at the final measured time point 52.5 h after plating (Fig. 4B). This distribution was consistent with random neurite sprouting that occurred at a rate obtained from the exponential fit in Fig. 4A and that was independent of neurite count (reduced *χ* ^2^ = 1.5).

**Figure 5:**
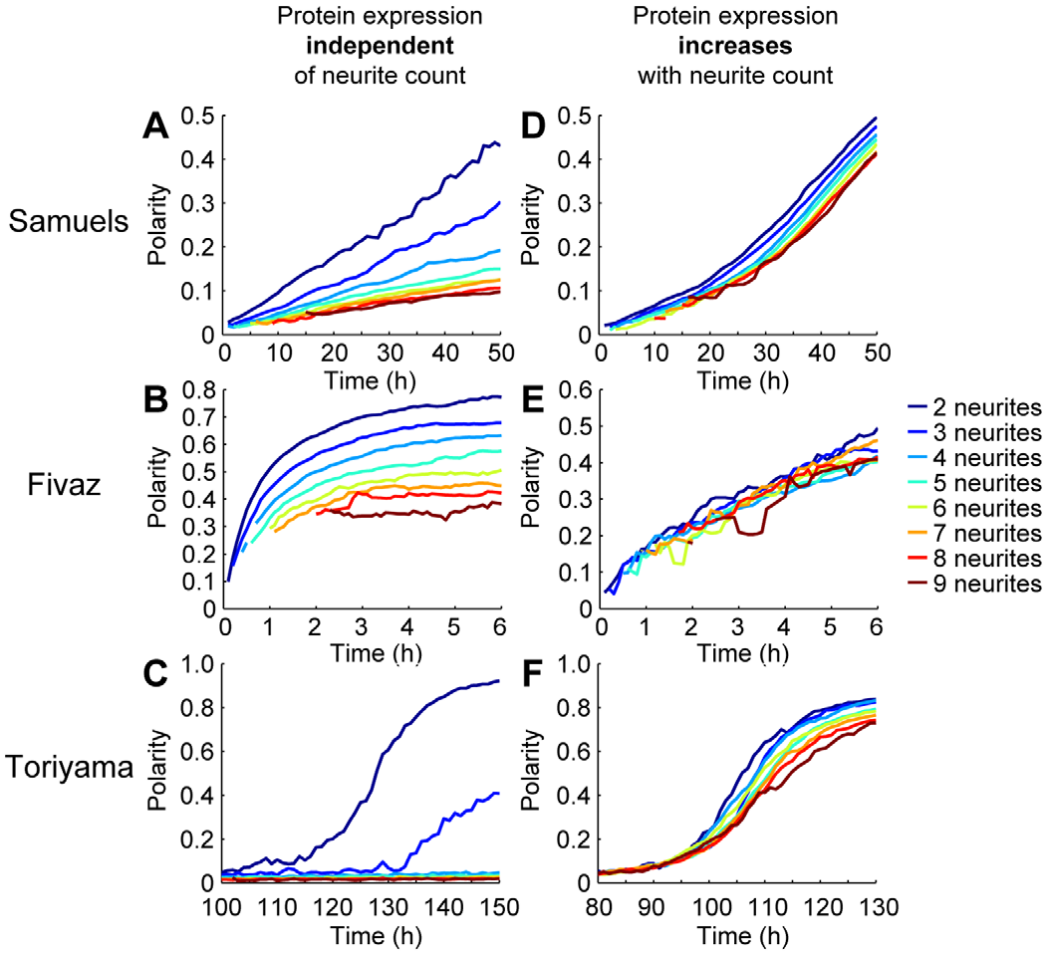
Polarity as a function of time and neurite count as predicted by the three models of neuronal symmetry breaking modified with dynamic neurite counts. Continuous curves were generated by connecting data for each neurite count at different time points. **A–C**, Polarity vs. time curves for different neurite counts in the Samuels, Fivaz, and Toriyama models with dynamic neurite counts. In the Fivaz model, HRas expression was normalized so that it was independent of neurite count, as in Fig. 3E. **D–F**, Expression levels of the protein underlying symmetry breaking now increases linearly with neurite count in all three models. In the Fivaz model, this protein is HRas; in the Toriyama model, it is shootin1; and in the Samuels model, “protein” refers to the rate-limiting chemical for neurite growth. Further details on how the Samuels and Toriyama models were modified can be found in the Materials and Methods section.

We then modified the Samuels, Fivaz, and Toriyama models by including this “dynamic neurite count” (see Materials and Methods section for further details), thereby making them more biophysically accurate. The addition of a dynamic neurite count increased the polarity of neurons with many neurites in the Samuels model (Fig. 5A) and in the version of the Fivaz model in which HRas expression was independent of neurite count (Fig. 5B) (compare with Figs. 3B and 3E). However, polarization among neurons with different neurite counts remained asynchronous, as the separation between the polarity vs. time curves remained significant. Adding a dynamic neurite count also had little effect on the Toriyama model (Fig. 5C), except to universally delay symmetry breaking in all neurons (compare with Fig. 3D). In summary, we found that a dynamic neurite count was insufficient for explaining the synchronous polarization behavior we observed in neurons with different neurite counts.

### Increased Expression of HRas/shootin1 in Neurons with More Neurites can Explain Synchronous Polarization among Neurons with Different Neurite Counts

Among the Samuels, Fivaz, and Toriyama models with both fixed and dynamic neurite counts, the only potential explanation we found for the synchronous polarization of neurons with different neurite counts was that the HRas expression levels in the Fivaz model increased with neurite count. In the Fivaz model, neurite growth and polarization rates ultimately depend on HRas concentration by way of several positive feedback loops, and so an increase in HRas levels can accelerate neurite growth and axon specification.

The Toriyama model consists of similar feedback loops that depend on shootin1 expression levels. We hypothesized that if shootin1 expression levels increased with neurite count in the Toriyama model, and similarly that if expression levels for the rate-limiting chemical for neurite growth increased with neurite count in the Samuels model, then both of these models might also agree with our experimental observations in Fig. 3A. The results of these modifications are shown in Figs. 5D and 5F, as well as the Fivaz model with a dynamic neurite count in Fig. 5E (further details of these modifications can be found in the Materials and Methods section). In Figs. 5D–F, the polarity vs. neurite count curves are all closely bundled together. Thus, by coupling the expression level of the protein underlying symmetry breaking (e.g., HRas, shootin1, or some other rate-limiting protein for neurite growth) to neurite count, all three models become consistent with our experimental finding that neurons with different neurite counts polarize synchronously.

### HRas and Shootin1 Expression Levels Increase with Neurite Count

**Figure 6:**
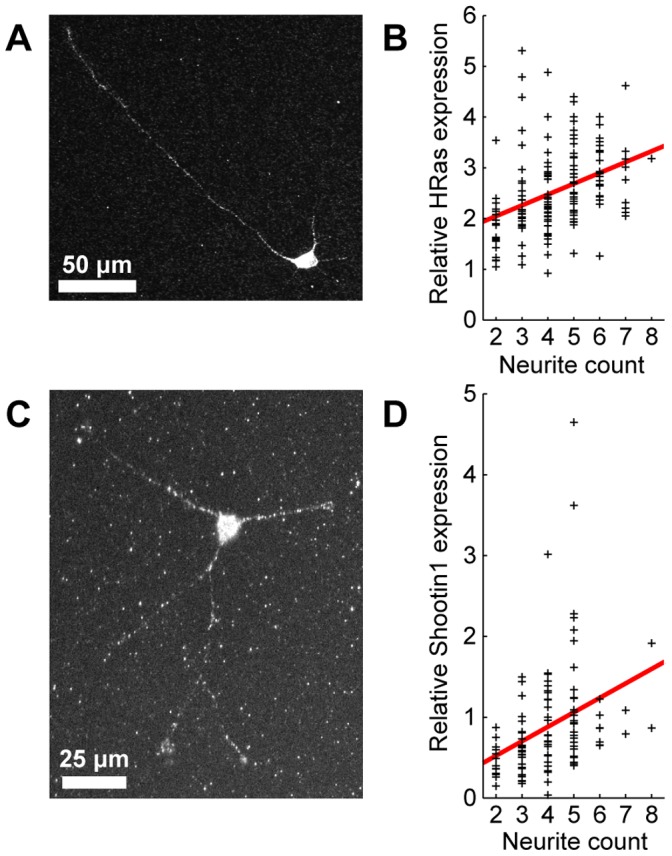
HRas and shootin1 expression in developing hippocampal neurons as a function of neurite count after 40 h in culture. **A**, Typical immunocytochemical stain for HRas in a neuron with 5 neurites. **B**, Typical immunocytochemical stain for shootin1 in a neuron with 3 neurites. The brightness in both **A** and **B** were saturated to make the neurites more visible. When these images were analyzed to determine relative HRas and shootin1 expression levels, image brightness was kept unsaturated. **C**–**D**, HRas/shootin1 expression as a function of neurite count. Individual neurons are indicated by plus signs, while the solid line indicates linear fits to the data. In both trend lines, the slope was significantly positive (*p* < 10^−4^ for both fits).

Finally, we tested our hypothesis that HRas and/or shootin1 expression levels increase with neurite count. To do this, we performed immunocytochemical stains of hippocampal neurons cultured for 40 h, and then calculated the relative fluorescence of approximately 150 neurons for each stain, recording neurite count as well. Figs. 6A–B show sample stains for HRas and shootin1, respectively. We found that both HRas and shootin1 expression significantly increased with neurite count (Figs. 6C–D, see Table S2 for more information).

Since we found that both HRas and shootin1 expression levels increase with neurite count, and that neurite count increases with time, we finally determined whether expression of these proteins also increases with time. Toriyama et al. previously showed via a western blot that shootin1 levels increase by approximately an order of magnitude over the first 24 hours after plating [18]. We performed a similar blot, instead labeling HRas, and we found that HRas expression significantly increases over the first 12 hours after plating the neurons (Fig. S2).

## Discussion

Here we have examined, for the first time, the role of neurite count in neuronal symmetry breaking. To do this, we introduced a new metric for neuronal polarity that uses information about all of a neuron’s neurites, unlike previously used metrics [10], [20]–[22]. Other metrics that meet this criterion are possible – for example, one might construct a polarity metric based on Shannon entropy, treating the relative lengths of the neurites as discrete probabilities. However, the metric we used is relatively simple, captures the phase transition as neurons polarize (Fig. 1B), and successfully illustrates the similarities and differences among various models and our experimental data. Furthermore, because of our metric’s linear nature, it is better suited for measuring small changes in polarity when neurons are highly unpolarized.

Previous experiments have qualitatively shown that neurons typically polarize 24–48 h after plating [5]. While the average polarity, as defined by Eq. (1), was found to monotonically increase over all the time points observed (as early as 7.5 h after plating), our results do not contradict previous findings. Because of the sensitive nature of our polarity metric, we were able to detect minor shifts in polarity in neurons at early times points with as little as 2 or 3 neurites, although these neurons were not unambiguously polarized to the naked eye. We found that the majority of neurons achieved a polarity of 0.3 only after 48 h. After 12 hours, 26% of the neurons we imaged had a polarity greater than 0.3. By 40 h, this percentage had increased to 48%, and after 52.5 h it was 57%. Thus, while polarization visibly occurs 24–48 h after plating, we have shown that symmetry breaking (as defined by growing differences among lengths of neurites, resulting in an increased polarity according to our metric) indeed begins at earlier time points.

We then applied our metric to study whether neurons with different neurite counts polarize at different times. Since a neuron with more neurites should have more degrees of freedom during symmetry breaking, one may expect to find that neurons with more neurites take longer to polarize. For instance, Toriyama et al. reported that their model predicts a 24 h temporal lag between symmetry breaking in neurons with 3 and 6 neurites [18]. In both the Samuels and Toriyama models, we found that neurons with 10 neurites polarize several days later than neurons with 2 or 3 neurites do. However, previous experiments had shown that neurons with various final neurite counts all polarize within the same 48 h time window [3]–[5]. Our experiments, which were conducted with a temporal resolution of 6 h, were consistent with these findings: we found no time delay in the polarization of neurons with different neurite counts, and that they instead broke symmetry synchronously. Such coordinated symmetry breaking may be important for synchronized polarization of neurons in developing organisms.

The leading models agree that additional neurites should slow symmetry breaking when the expression of specific proteins (e.g., HRas in the Fivaz model, shootin1 in the Toriyama model, or some other rate-limiting protein for neurite growth in the Samuels model) is independent of neurite count. We have shown that increased expression of these proteins accelerates symmetry breaking in each model. This acceleration occurs because the models include these proteins in positive feedback loops, so that increases in their intracellular concentration speed up the entire polarization process. When the expression of these proteins increases with neurite count, the two effects (increased protein expression accelerating symmetry breaking, a higher neurite count decelerating symmetry breaking) can cancel out, so that neurons with different neurite counts break symmetry at the same rate, consistent with our experimental results.

Such a relation between protein expression and neurite count does not inherently contradict the previous experimental findings of Fivaz et al. or Toriyama et al. The Fivaz model already assumed that HRas expression and neurite count were proportional, while Toriyama et al. measured overall shootin1 expression in bulk, using 1.7×10^5^ neurons, and so it is possible that shootin1 expression varied with neurite count in their experiments. Here, we measured HRas and shootin1 expression levels in several hundred developing neurons individually, and found that the expression of both proteins increased significantly with neurite count.

Future work might examine the causality of these trends, i.e., whether a higher neurite count increases HRas/shootin1 expression, or whether higher HRas/shootin1 expression levels increase neurite count. HRas and shootin1 overexpression have been previously shown to induce the formation of supernumerary axons [9], [10], [18], but have not yet been shown to affect neurite count. Alternatively, both HRas/shootin1 expression levels and neurite sprouting could be controlled by some other agent, such as intrinsic proteins (e.g., Rho GTPases, the PAR complex, etc.) or extrinsic signaling [13].

Thus, while additional neurites could hinder symmetry breaking, our results suggest that neurons can and do overcome this via increased expression levels of their symmetry-breaking machinery (e.g., HRas, shootin1, etc.), allowing neuronal symmetry breaking among neurons with different neurite counts to occur synchronously.

## Materials and Methods

### Neuron Isolation and Culture

Primary hippocampal neurons from embryonic day 18 (E18) Sprague-Dawley rats were used for both time-lapse studies. All animal work was approved by the MIT Committee for Animal Care (which performs annual reviews of all animal protocols) and the Division of Comparative Medicine, and abides by all institutional, state, and federal guidelines for animal welfare. Timed pregnant female rats were purchased from Charles River Laboratories. To maximize consistency for embryo age, tissue harvesting was always performed at the same time of day. The rats were euthanized by CO_2_ asphyxiation, followed by cervical dislocation. Male and female E18 rat embryos were removed from the mother, and decapitated with sharp scissors. After decapitation, the embryo’s skull was cut and removed prior to scooping out the brain and placing in ice-cold HEPES buffered HBSS in a petri dish surrounded by ice. Meninges were removed prior to dissecting the hippocampi.

Hippocampi were dissected and placed in ice-cold Hank’s balanced salt solution (HBSS) buffered with 10 mM HEPES at pH 7.3. The hippocampi were then transferred to and mixed in a solution containing 20 units of papain per ml of HBSS, 1 mM CaCl_2_, and 1 mM L-cysteine, and then incubated at 37°C for 30 min. The cells were subsequently washed in Neurobasal-B27 (Invitrogen) containing 2 mM glutamine and 100 units/ml penicillin/streptomycin.

For both time-lapse imaging and immunocytochemical studies, prior to cell plating, several wells of two 96-well plates (Matrical) were incubated in 10 µg/ml poly-D-lysine (PDL) in phosphate buffered saline (PBS) overnight at room temperature, rinsed with PBS, incubated in 10 µg/ml laminin in PBS for 1 h at 37°C, and finally rinsed again with PBS. Neurons were plated at a density of ∼1000 cells/well in 300 µl of the Neurobasal-B27 media. For the western blot, 1 million cells were plated onto 40 mm glass-bottom dishes that were similarly coated with PDL and laminin.

### Time-lapse Imaging

For the time-lapse experiment, the locations of 200 randomly chosen neurons were recorded after 3.5 h *in vitro* so that subsequent images of these neurons could be taken quickly. A total of nine time points of images were taken, at 7.5, 12, 17, 22.5, 28.5, 34.5, 40.5, 46.5, and 52.5 h after the neurons were plated. Neurons were discounted if no neurites grew out over the two days, or if there was significant crossing of the neurites. Time points from specific neurons were also discounted if their neurites came into contact with the neurites of neighboring neurons such that the neurites of two neurons could not be distinguished. Of the 200 neurons, 132 were used for further analysis.

Images were captured using a 20× objective (NA 0.75, Nikon) and a CoolSnap HQ2­_­_ CCD camera via bright field microscopy. To enhance the contrast of the neurons’ boundaries, an adaptive histogram equalization algorithm (*adapthisteq* in MATLAB v7.12) was applied to the images. Skeletons of the neurites were identified manually. When a neurite branched, the longest branch was used in measuring the neurite’s length.

### Neuronal Polarity Metric

Previously, the most commonly used polarity metrics have included the absolute [20]–[22] or relative length of a neuron’s longest neurite [10]. However, these definitions have several flaws. Using the absolute length of the longest neurite discards significant information regarding the lengths of the remaining neurites. For example, if a neuron’s longest neurite is 150 µm, one cannot say whether it is highly polarized: it may be polarized if the second-longest neurite is 30 µm, but not if the second-longest neurite is 120 µm. Using the relative length of the longest neurite (i.e., the ratio between the length of the longest neurite and the total neurite length) poses similar problems. By this definition, a neuron with exactly two neurites must have a polarity exceeding 0.5, while a neuron with many neurites has a wider range of polarities, making comparisons between neurons with different neurite counts difficult. Moreover, a neuron with ten neurites that are 10 µm long and a single neurite that is 100 µm long will have the same polarity as a neuron with two 20 µm neurites and one 40 µm neurite. While common sense suggests that the former neuron is more polarized, this definition produces equal polarities because it too discards significant information regarding the shorter neurites.

We previously introduced a quantitative metric for polarity for neurons with two neurites [19]. If these neurites have lengths *L*_1_ and *L*_2_, we defined the polarity, *P*_2_, as
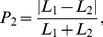
(2)a metric that ranges from zero (completely unpolarized, neurites are equal in length) to unity (completely polarized, one neurite is much longer than the other). We have previously experimentally shown that this metric displays a phase transition in neurite polarization as a function of neurite length as one would expect [19].

Here, we generalized Eq. (2) to neurons with more than two neurites. For a neuron with *N* neurites of length *L_1_*, …, *L_N_*, we define *x_i_* as the normalized length of neurite *i*:
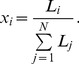
(3)


We then define the polarity using Eq. (1). The normalizing coefficient *N*/[2(*N*−1)] constrains the polarity between 0 and 1, and in the case of *N* = 2, Eq. (1) reduces to Eq. (2).

### Immunocytochemistry

A second batch of E18 hippocampal neurons was cultured for 40 h and then fixed for 30 min in 4% paraformaldehyde, and then washed twice using PBS with 0.05% Tween-20 (PBST). The neurons were then permeabilized for 10 min with 0.1% Triton X-100, and washed again in PBST. The surface was next blocked for 30 min with 3% bovine serum albumin (BSA) in PBS. The cells were then incubated for 60 min in primary antibodies: either mouse anti-HRas monoclonal antibody (Millipore) or rabbit anti-shootin1 polyclonal antibody (Pierce). After another wash in PBST, the secondary antibodies were applied for another 60 min: cells stained with mouse anti-HRas were then stained with goat anti-mouse AlexaFluor 555 (Invitrogen) and goat anti-rabbit AlexaFluor 546 (Invitrogen). Both secondary antibodies were visible using a Cy3 filter cube. The cells were again washed in PBST a final time.

Immunostained neurons were imaged using the same objective and camera used for the time-lapse recordings. A total of 156 HRas-stained neurons and another 111 shootin1-stained neurons were selected for analysis. Neurons were chosen if they had a clearly defined neurite count and they were not in contact with other neurons. The relative amount of HRas or shootin1 in each of the neurons was determined by cropping a polygon surrounding the entire neuron, including its cell body and all of its neurites, from the fluorescence image. The background pixel brightness was multiplied by the area of the cropped image, and this quantity was then subtracted from the total integrated brightness of the cropped image. Fluorescence images were also appropriately normalized to account for any inhomogeneities in the periphery of the focal plane due to optical aberrations.

A third batch of E18 hippocampal neurons was cultured for 40 h and similarly stained for tau1 and MAP2, which are axonal and dendritic markers, respectively [23]. For tau1, the primary antibody used was mouse monoclonal (MAB3420, Millipore). For MAP2, the primary antibody used was rabbit polyclonal (AB5622, Millipore).

### Western Blot

A Western blot for HRas expression was performed using 5×10^5^ neurons at three time points: 3, 7, and 12 h after plating. Cells were lysed using a mammalian cell lysis kit (Sigma), and the lysate was then concentrated using centrifugal filter units (Amicon Ultra 10K, Millipore). The concentrated lysate was run on an SDS-PAGE (4–12% Bis-Tris, Invitrogen), and then transferred to a nitrocellulose membrane under semi-dry conditions. The primary antibody used for the western blot was Rabbit polyclonal to HRas (ab97488, AbCam), and the secondary antibody was tagged with a quantum dot (WesternDot 625 goat-anti-rabbit, Invitrogen) that fluoresced under UV. A UVP GelDoc-It Imager was used for imaging the blot.

### Computational Modeling of Neurite Sprouting and Growth

For each of the models we analyzed, 5% Gaussian noise was added to all initial conditions, consistent with the initialization routine of Fivaz et al. [10]. When neurite count was dynamic, the neurite count was initialized to one, and new neurites were added to the model with an exponentially decaying probability, an initial length of 5 µm, and zero concentration of all model-specific molecules (e.g., HRas, shootin1, etc.), except where otherwise noted. The amplitude for this exponential sprouting rate was based on the fit to our experimental data, which had an asymptotic neurite count of 8 (see Fig. 2B). The time constant was similarly based on our experimental measurement of approximately 32 h, but was scaled to the model-specific simulation durations.

Due to inherent differences among the models, the simulation duration for each model was set so that the polarity of neurons with exactly two neurites achieved a polarity as defined by Eq. (1) of approximately 0.5. This ensured that, independent of other model parameters that may affect polarization rate, we could compare the effect of neurite count on polarization dynamics. The Samuels model was run for 50 h, the Fivaz model for 6 h, and the Toriyama model for 130 h (or 150 h when neurite count was dynamic but shootin1 expression was independent of neurite count). For all three models, neurites were initialized to a length of 5 µm, prior to adding Gaussian noise. The Samuels model was solved using the MATLAB ordinary differential equation solver *ode45*, while the more complex Fivaz and Toriyama models were solved using the MATLAB function *ode15s*.

The Samuels model consists of three dimensionless parameters, which were set to values that induce axon specification (*χ*_1_ = 5, *χ*_2_ = 100, and *χ*_3_ = 5), as well as a characteristic length (set to 50 µm) and time, which was set to either 20 h (when neurite count was fixed) or 80 h (when neurite count was dynamic) so that the different simulations broke symmetry on similar timescales and could be compared [17]. Prior to Gaussian noise, the initial non-dimensionalized concentration of the rate-limiting protein in the cell body was set to 1/*χ*_2_, its steady-state value, and the concentration in each neurite tip was set to 1 divided by the neurite count (the steady-state value when there is no axon specification). Protein expression was modeled as being proportional to neurite count by modifying the original equation of the Samuels model for expression of the rate-limiting chemical for neurite growth in the cell body:
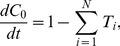
(4)where *C*_0_ is the normalized concentration in the cell body, *N* is the neurite count, and *T_i_* is the active transport rate to neurite *i*, so that it became:



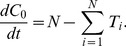
(5)For the Fivaz model, all parameters were set equal to those originally proposed [10]. Fivaz and coworkers initialized the quantity of HRas and phosphatidylinositol-3,4,5-triphosphate in each neurite to a constant value, resulting in an approximately linear relationship between neurite count and HRas quantity [10]. In modeling HRas expression as independent of neurite count, we normalized the initial HRas and phosphatidylinositol-3,4,5-triphosphate (PIP_3_) concentrations so that the total quantity of HRas in the cell body and neurite tips remained constant for each neurite count, and sprouting neurites were initialized with zero HRas and PIP_3_ so that expression levels remained fixed when neurite count was dynamic.

The Toriyama model was simulated largely as originally described [18]. However, we were unable to produce axons using the original equation for the somatic HRas concentration, *S*:
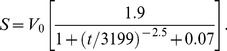
(6)


In this equation, *V*_0_ is the volume of the cell body, and *t* is time in minutes. To produce neurons with single axons, we increased the numerator in the time-dependent term by approximately 50 percent, to 2.7:
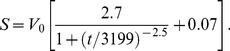
(7)


This model is unique among the three in that symmetry breaking is not evident until dozens of hours into the simulation, even when only two neurites are present. For modeling protein expression that increases with neurite count, we modified this equation similarly to how we modified the Samuels model, i.e., by adding a linear term that depends on neurite count *N*:
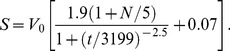
(8)


## Supporting Information

Figure S1 **Fluorescent immunocytochemical stain for axonal markers 40 h after plating.** **A**-**E** are five different micrographs of representative neurons. Tau1, an axonal marker, is shown in green, while MAP2, a dendritic marker, is shown in red. Nuclei were stained with DAPI, and are shown in blue.
(TIF)

Figure S2 **Western blot for HRas in developing neurons during the first 12 h after plating.** HRas was immunoblotted using a polyclonal antibody 3, 7, and 12 h after plating. Relative HRas expression was quantified by integrating the 20 kDa bands in each lane.
(TIF)

Table S1 **Summary of the number of neurons and neurite data at each time point.**
(DOC)

Table S2 **Summary of the number of neurons and relative expression levels of HRas and shootin1 as determined by immunocytochemistry.**
(DOC)
