# Supplementary figures and images for: Synchronous Symmetry Breaking in Neurons with Different Neurite Counts
Source: PLoS One. 2013 Feb 11;8(2):e54905. doi: 10.1371/journal.pone.0054905 (PMC3569465; doi:10.1371/journal.pone.0054905)

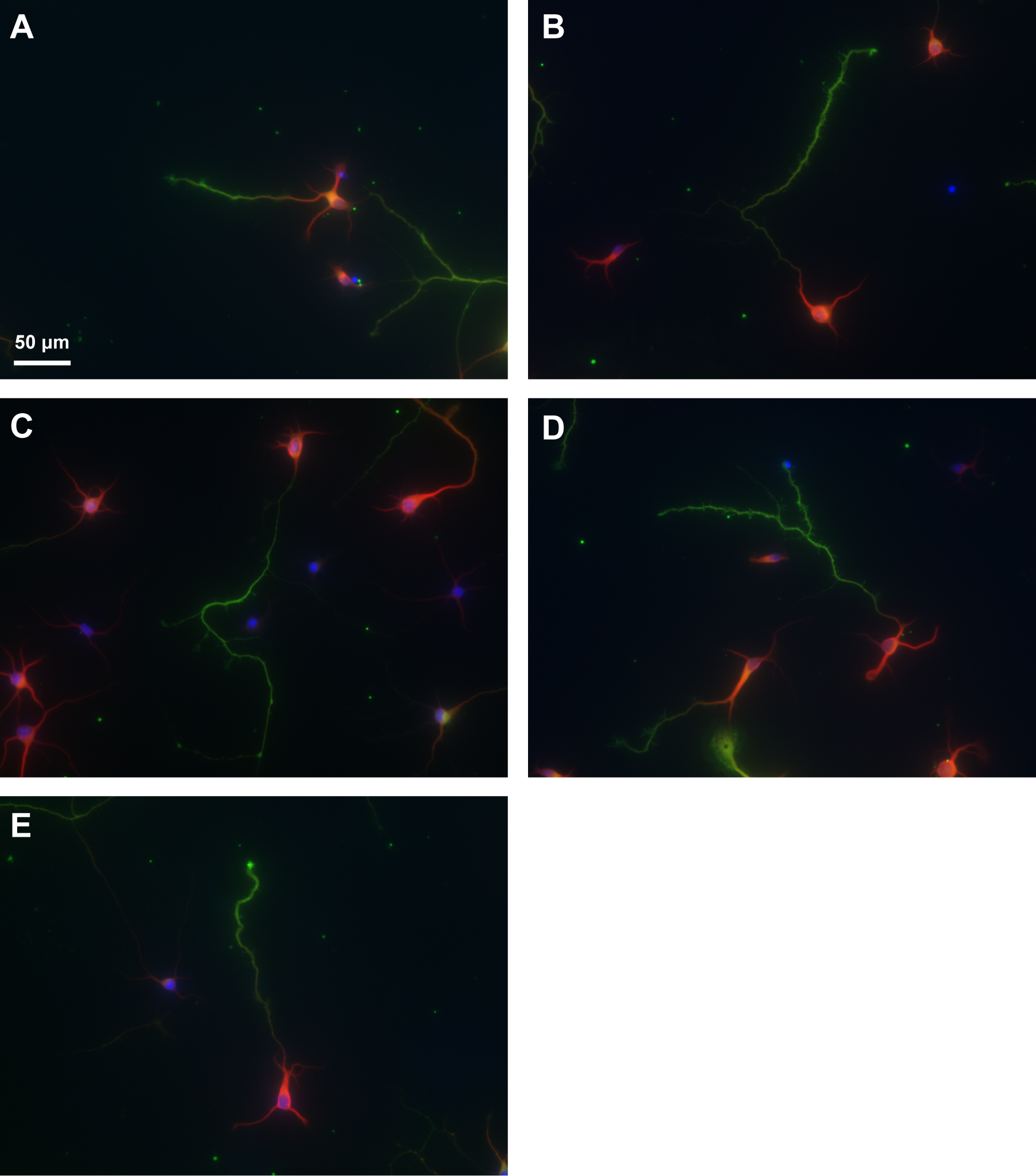

Supplement: Figure S1 — Fluorescent immunocytochemical stain for axonal markers 40 h after plating. A-E are five different micrographs of representative neurons. Tau1, an axonal marker, is shown in green, while MAP2, a dendritic marker, is shown in red. Nuclei were stained with DAPI, and are shown in blue. (TIF) [file pone.0054905.s001.tif]

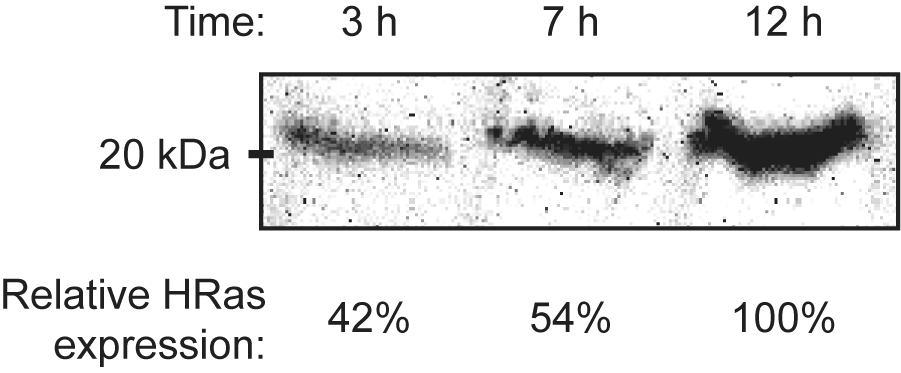

Supplement: Figure S2 — Western blot for HRas in developing neurons during the first 12 h after plating. HRas was immunoblotted using a polyclonal antibody 3, 7, and 12 h after plating. Relative HRas expression was quantified by integrating the 20 kDa bands in each lane. (TIF) [file pone.0054905.s002.tif]
