# Supplementary material for: Synchronous Symmetry Breaking in Neurons with Different Neurite Counts
Source: PLoS One. 2013 Feb 11;8(2):e54905. doi: 10.1371/journal.pone.0054905 (PMC3569465; doi:10.1371/journal.pone.0054905)
Supplement: Table S1 — Summary of the number of neurons and neurite data at each time point. (DOC) [file pone.0054905.s003.doc]

| **Time point (h)** | **Number of neurons** | **Neurite count (mean ± SE)** | **Polarity (mean ± SE)** |
| --- | --- | --- | --- |
| 7.5 | 70 | 2.94 ± 0.13 | 0.217 ± 0.017 |
| 12 | 94 | 3.29 ± 0.13 | 0.233 ± 0.015 |
| 17 | 111 | 4.11 ± 0.16 | 0.237 ± 0.013 |
| 22.5 | 113 | 4.68 ± 0.19 | 0.248 ± 0.013 |
| 28.5 | 122 | 4.96 ± 0.21 | 0.253 ± 0.014 |
| 34.5 | 125 | 5.40 ± 0.22 | 0.289 ± 0.015 |
| 40.5 | 125 | 5.82 ± 0.24 | 0.295 ± 0.015 |
| 46.5 | 124 | 6.10 ± 0.25 | 0.313 ± 0.014 |
| 52.5 | 122 | 6.60 ± 0.25 | 0.341 ± 0.015 |
