# Supplementary material for: Synchronous Symmetry Breaking in Neurons with Different Neurite Counts
Source: PLoS One. 2013 Feb 11;8(2):e54905. doi: 10.1371/journal.pone.0054905 (PMC3569465; doi:10.1371/journal.pone.0054905)
Supplement: Table S2 — Summary of the number of neurons and relative expression levels of HRas and shootin1 as determined by immunocytochemistry. (DOC) [file pone.0054905.s004.doc]

|  | **HRas** | | **Shootin1** | |
| --- | --- | --- | --- | --- |
| **Neurite count** | **Number of neurons** | **Relative expression (mean ± SE)** | **Number of neurons** | **Relative expression (mean ± SE)** |
| 2 | 19 | 1.81 ± 0.13 | 14 | 0.46 ± 0.05 |
| 3 | 26 | 2.52 ± 0.20 | 30 | 0.63 ± 0.06 |
| 4 | 42 | 2.37 ± 0.11 | 26 | 0.93 ± 0.12 |
| 5 | 35 | 2.79 ± 0.13 | 28 | 1.28 ± 0.19 |
| 6 | 24 | 2.94 ± 0.12 | 7 | 0.85 ± 0.08 |
| 7 | 9 | 2.84 ± 0.27 | 2 | 0.94 ± 0.15 |
| 8 | 1 | 3.18 | 2 | 1.4 ± 0.5 |
